# Brain-to-Brain Synchrony during Naturalistic Social Interactions

**DOI:** 10.1038/s41598-017-17339-5

**Published:** 2017-12-06

**Authors:** Sivan Kinreich, Amir Djalovski, Lior Kraus, Yoram Louzoun, Ruth Feldman

**Affiliations:** 1 Gonda Brain Sciences Center, Bar-Ilan University, Ramat Gan, Israel; 2 Department of Psychology, Bar-Ilan University, Ramat Gan, Israel; 3 Department of Mathematics, Bar-Ilan University, Ramat Gan, Israel; 4 Baruch Ivcher School of Psychology, Interdisciplinary Center, Herzlia, Israel; 5 Yale University Child Study Center, New Haven, USA

## Abstract

The evolution of humans as a highly social species tuned the brain to the social world; yet the mechanisms by which humans coordinate their brain response online during social interactions remain unclear. Using hyperscanning EEG recordings, we measured brain-to-brain synchrony in 104 adults during a male-female naturalistic social interaction, comparing romantic couples and strangers. Neural synchrony was found for couples, but not for strangers, localized to temporal-parietal structures and expressed in gamma rhythms. Brain coordination was not found during a three-minute rest, pinpointing neural synchrony to social interactions among affiliative partners. Brain-to-brain synchrony was linked with behavioral synchrony. Among couples, neural synchrony was anchored in moments of social gaze and positive affect, whereas among strangers, longer durations of social gaze and positive affect correlated with greater neural synchrony. Brain-to-brain synchrony was unrelated to episodes of speech/no-speech or general content of conversation. Our findings link brain-to-brain synchrony to the degree of social connectedness among interacting partners, ground neural synchrony in key nonverbal social behaviors, and highlight the role of human attachment in providing a template for two-brain coordination.

## Introduction

Humans are fundamentally socind the capacity to function competently within the social world shapes our physical health and emotional well-being throughout life^1–3^. The human brain, immature at birth and relying on the sensitive caregiving of a social agent, has evolved to support life within the social milieu, constantly receiving information, updating predictions, assing communicative intents, interpreting interactive signals, and responding dynamically to the social world^4^. Recent models in social neuroscience have called to move from a solipsistic to a “situated” perspective on brain functioning and to formulate novel neuroscience frameworks that can accommodate the inherently social nature of the human brain^5–7^. As a first step for such two-person neuroscience, there is a need to define mechanisms that enable two humans to coordinate their brain response online during social interactions. Yet, the greatest challenge in two-brain research is ecological validity^6^. To date, most studies on two interacting brains utilized unnatural settings, such as human-avatar or human-computer interactions^8^ or two individuals lying in separate fMRI^9^ or MEG machines^10^, settings where the entire social envelop is altered. No study, to our knowledge, has tested correlations between EEG activations recorded simultaneously from two adults during naturalistic interaction in relation to ongoing social behavior. Thus, the goal of the current study was to examine whether natural social moments induce linked EEG activations between two brains and whether the degree of social connectedness among partners may play a role in such linkage.

The coordination of behavior between two or more individuals– behavioral social synchrony (called hereafter “social synchrony” and implying some pattern of behavior coordination) – is a fundamental aspect of social life^11–14^. Social synchrony is an evolutionary-ancient mechanism that binds members into a social group; rodents^15^ and primates^16,17^ exhibit behavioral mimicking, a precursor of human social synchrony, and in both, familiarity with conspecific bolsters behavioral matching^18^. Across mammalian species, social synchrony is learned within the mother-infant bond through processes of *bio-behavioral synchrony*, the coupling of parent and infant’s physiology and behavior during moments of social contact, and, thus, attachment contexts provide the arena for the experience and encoding of synchrony^19^. Episodes of social synchrony between parents and infants carry profound effects on the maturation of physiological systems that support participation in social life. For instance, during episodes of social synchrony in the gaze and affect modalities there is also a coupling of parent and infant’s heart rhythms^20,21^ and coordinated release of oxytocin^22^, suggesting that social synchrony provides a template for the emergence of biological synchrony between attachment partners.

Social synchrony experienced during early sensitive periods provides the foundation for the expression of synchrony in later attachment bonds throughout life^19,23^. Animal^24^ and human studies^25^ indicate that similar neural systems and behavioral patterns underpin the parent-infant and pair bonds and demonstrate that both forms of human attachment are characterized by behavioral synchrony^26–28^. During naturalistic interactions, romantic partners exhibit online coordination of gaze and affect patterns^9^ and an increase in oxytocin levels that correlates with the degree of social synchrony^28^. Thus, the search for mechanisms that enable two adults to coordinate their brain response in real life may profit from investigating natural social moments, focusing on romantic partners as a prototypical relationship that propagate synchrony, and anchoring neural synchrony in key nonverbal social behaviors that are learned within the first social dialogue between parents and infants, such as gaze and affect.

Brain areas that support brain-to-brain neural synchrony may involve temporal-parietal structures, including the posterior superior temporal sulcus (pSTS) and temporo-parietal junction (TPJ), and studies using a variety of methods have indeed pinpointed neural synchrony to these regions. These studies have also found that the degree of social connectedness among partners, as indexed by multiple factors such as familiarity, predictability, or collaboration, is associated with the level of neural synchrony. Dikker *et al*.^29^ found that brain coordination among two interacting individuals was observed in the pSTS pending on the predictability of the interaction^30^. A hyper-scanning fNIRS study showed neural synchrony in the rTPJ during face-to-face, but not during face-blocked interaction, suggesting that social gaze may play a role in neural synchrony. Furthermore, the degree of neural synchrony was related to the level of shared intentionality among partners^31^. Neural synchrony of BOLD activations was found in parietal and temporal regions during an emotional exchange^9^; An fNIRS study found neural synchrony in the rTPJ when partners engaged in a cooperative task, but not during parallel play^32^; and the degree of neural synchrony was found to be associated with behavioral synchrony^33^. Overall, these studies indicate that the degree of social connectedness among partners impacts the level of neural synchrony in temporal-parietal structures. As behavioral synchrony is linked with attachment status, with couples and close friends expressing more synchrony than strangers^26,34^, it is reasonable to expect that romantic partners in a long-term relationship would display more behavioral and neural synchrony as compared to strangers and that neural synchrony would localize to temporo-parietal areas.

One mechanism that has been proposed to support neural synchrony is brain oscillations, particularly gamma-band oscillations (30–90 Hz) which have been implicated in socially-relevant functions^8^. Gamma activity recorded from temporal-parietal areas has been linked with a range of social abilities that support social connectedness, such as theory-of-mind, cognitive appraisal, and emotion regulation^8,35,36^. Kang *et al*.^35^, measuring event-related changes in gamma-band power, found greater parietal gamma in response to emotional, compared to neutral pictures and gamma involvement in social reappraisal processes. Increased intracranial neural oscillations in the gamma range localized to the pSTS were observed during a mentalizing task that required consideration of own versus another person’s perspective^8^. An animal study involving temporal and parietal cortices implicated gamma activity in the binding of sensory representations^37^ and, in humans, audiovisual looming signals elicited increased gamma-band coherence between auditory cortex and the STS^38^, suggesting that gamma oscillations may provide a fast-paced template for the coordination of two brains.

In light of the above, the current study used EEG hyperscanning to assess brain-to-brain synchrony during a natural social interaction between male and female adults. Our central hypothesis was that the degree of social connectedness among partners would impact the level of neural synchrony and thus, we compared long-term romantic couples and strangers. We also examined whether neural synchrony is grounded in behavioral coordination. Hyperscanning EEG is a recently-developed methodology for recording EEG simultaneously from two or more individuals engaged in a social task. It affords the recording of real-time neural dynamics from two brains, which can then be analyzed for inter-individual coupling without compromising ecological validity. As processes of biological and behavioral synchrony have been observed in the context of attachment relationships^3,39^, we expected greater brain-to-brain neural synchrony in couples compared to strangers (hypothesis 1), and hypothesized that neural synchrony would localize to temporal-parietal regions and express in gamma-band rhythms (hypothesis 2). In addition, we expected greater social synchrony in the gaze and affect modalities among couples compared to strangers (hypothesis 3). Consistent with research indicating that neural synchrony is impacted by the degree of social connectedness^29^ and that synchrony of heart rhythms between mother and child was observed during episodes of social gaze and positive affect^19^, we expected that moments of social gaze and positive affect, compared to episodes of no gaze and neutral affect would elicit greater neural synchrony (hypothesis 4). Finally, while less neural synchrony was expected in strangers compared to couples, we hypothesized that more social gaze and positive affect and greater sense of social connectedness in this group would correlate with higher neural synchrony (hypothesis 5).

## Results

Hyperscanning EEG recording was used to test brain-to-brain neural synchrony between 104 adults, comprising male-female pairs of cohabitating couples versus strangers. Dyads were videotaped and EEG-recorded during a free conversation about a positive theme. Interactions were micro-coded offline for gaze and affect. Artifact removal procedure was applied and percentage of epochs and electrodes participating in the final analyses appear in Supplementary Table 1.

### Brain-to-Brain Neural Synchrony

**Figure 1:**
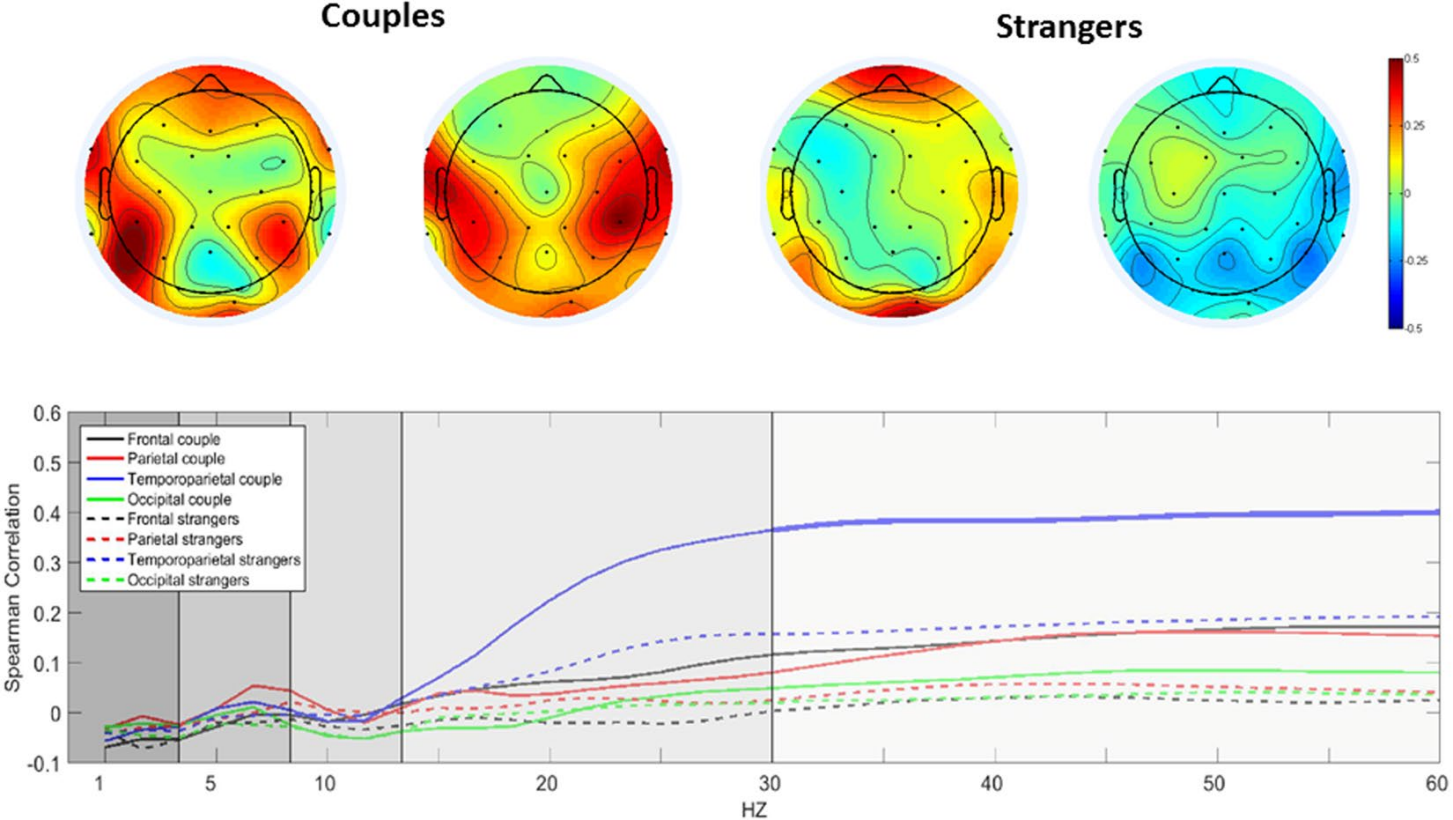
Top Panel: Example of a spatial distribution of gamma power correlations in one couple dyad and one stranger dyad over the entire scalp. Spearman correlation was applied over the continuous gamma power of similar channels for the male and female of each dyad. Correlations are shown separately for couples and strangers and reflect higher correlations in temporal-parietal areas for couples. Plots are constructed by mapping Spearman correlation using the function topoplot from EEGLab^66^. Bottom Panel: Dyadic Correlation Spectral analysis. The continuous Fourier transform of each EEG electrode (Stockwell transform) was averaged across ROIs (occipital, frontal, parietal, and temporoparietal) and correlated with the partner’s. Graph shows the correlations values for every frequency bin averaged across groups (couples, strangers). Significant correlation values were found across the gamma frequency (30–60 HZ) over the temporal-parietal area for couples (Thick blue line).

EEG correlations were used to test frequencies and localization of brain-to-brain neural synchrony. A full spectral dyadic correlation analysis was conducted including the entire scalp divided into four ROIs (frontal, occipital, parietal, and temporoparietal). The power spectra divided by frequency bands was calculated for each electrode, averaged over electrodes for each ROI. The temporal pattern of the spectrum was then correlated with the respective averaged power of the partner (Fig. 1 bottom panel).

The averaged Spearman correlations of the recorded EEG power spectrum across all dyads averaged over the two groups appears in Fig. 1. The distribution of the frequencies (1–60 Hz) averaged over the various bands revealed significant correlation in gamma power (30–60 Hz) between interacting couples averaged across electrodes located in the temporal/parietal area, R = 0.389, p = 0.0028. In contrast, gamma correlation among interacting strangers was non-significant, R = 0.102, p = 0.10. Comparison between gamma correlations in couples and strangers indicated higher correlation in the couples compared to the strangers group, *t* (two-tailed) = −3.02, p = 0.004. The calculated tomographic LORETA images corresponded to the estimated neuronal generators of brain activity within the gamma frequency range^40^. Correlations of theta, alpha, and beta power did not reach significance in any ROI. These findings support our first and second hypotheses. Spatial distribution of the gamma correlation over the entire scalp for two partners from each group appears in Fig. 1 (Top panel) and illustrates the localization of neural synchrony over the temporoparietal area for couples, but not for strangers.

### Rest Analysis

Dyadic correlations in temporal-parietal gamma were calculated for the rest paradigm and compared among groups. We then computed the difference between gamma correlation at rest and gamma correlation during the interaction for each group. Gamma correlations during rest were non-significant for both couples, R = 0.16, p = 0.10, and strangers, R = 0.07, p = 0.10, with no difference between groups. Among couples only, gamma correlations increased significantly from rest to social interaction, paired-sample *t*-test, *t* (two-tailed) = 3.8801, p = 0.00075. Among strangers, no significant change was observed in gamma correlations between rest and social interaction, *t* (two-tailed) = 1.779, p = 0.0878. These findings lend further support to our hypothesis that the neural synchrony found in couples is specific to social interactions and that the degree of social connectedness plays a role in neural synchrony.

### Neural Synchrony and couples’ relationships

The correlations between partners’ temporal-parietal gamma were correlated with the two attachment variables from the Attachment in Close Relationship (ECR-R) questionnaire separately for males and females. A negative correlation was found for attachment anxiety in males, indicating that the greater the man’s attachment anxiety the lower the temporal-parietal gamma correlation during social interaction; Men; Spearman R = −0.3960, p = 0.0307, Women; Spearman R = −0.26, p = 0.228.

### Behavioral Measurement

#### Nonverbal Social Behavior: Gaze and Affect

**Figure 2:**
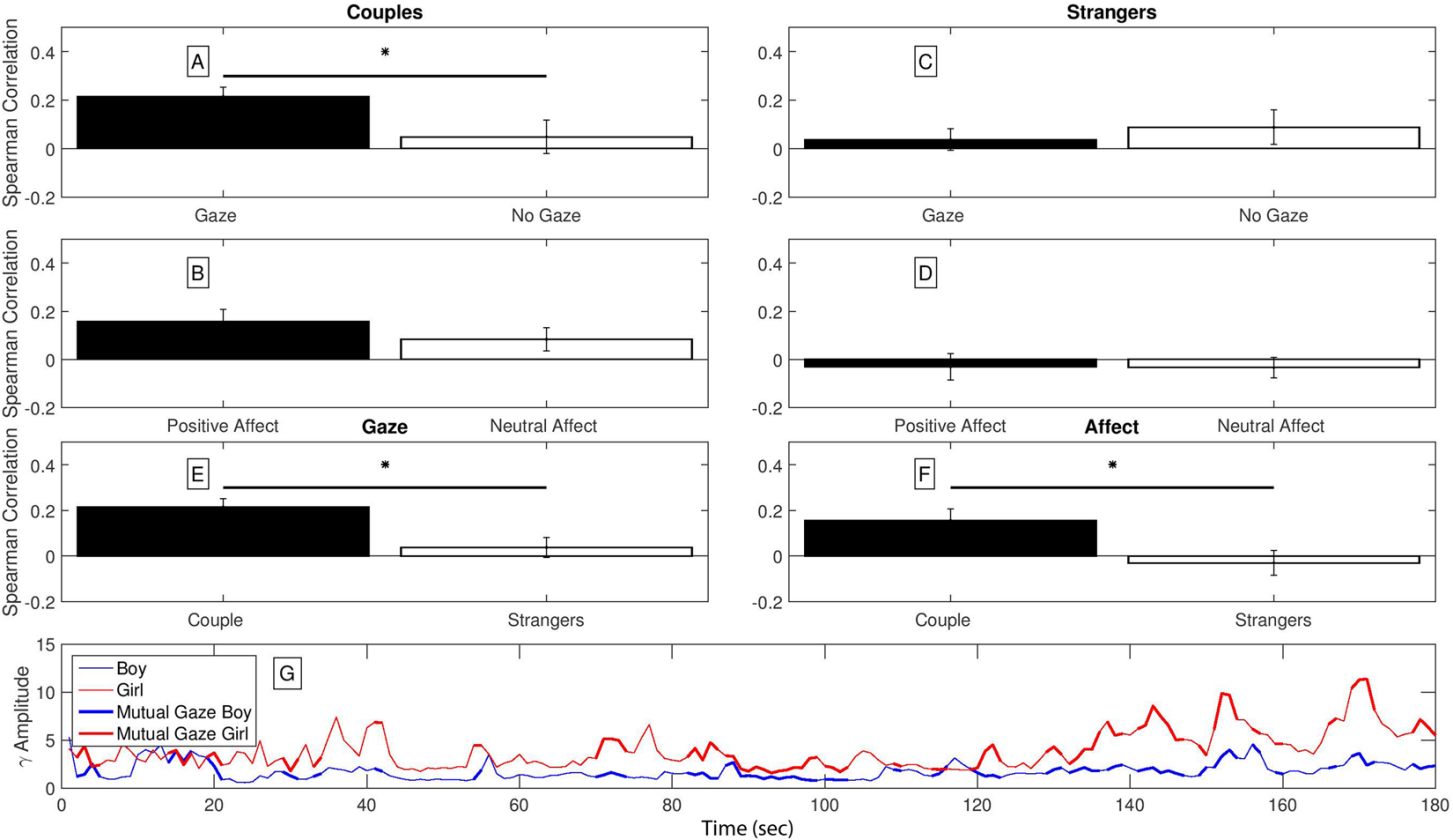
Top Panel: Dyadic gamma correlation values during episodes of social gaze and positive affect. Comparison of the averaged correlation between the partners’ temporal-parietal gamma power fluctuation during episodes of social gaze versus no-gaze and episodes of positive affect versus neutral affect for couples (**A**,**B**) and strangers (**C**,**D**). Higher neural correlation values emerged for couple pairs during episodes of social gaze (A, two-tailed t-test, p = 0.05). Bars represent mean and standard errors. Number of participants in each analysis: *Strangers;* social gaze (n = 25), no gaze (n = 11), positive affect (n = 23), no affect (n = 20). *Couples*; social gaze (n = 24) no gaze (n = 6), positive affect (n = 21), no affect (n = 19) (**E,F**). Direct comparison between temporal-parietal gamma power correlation in couples (n = 24) and strangers (n = 25) during episodes of social gaze and positive affect showed significant difference in the averaged correlation. Bars represent mean and standard errors. Social Gaze (two-tailed t-test, p = 0.0036), Positive Affect - (two-tailed t-test, p = 0.015). Bottom panel; Example of gamma power oscillation in one couple. Gamma power oscillation of one couple as it fluctuates during the interaction. The gamma power is calculated from the electrodes located in the same temporal-parietal area for both female and male. For presentation only, times of mutual gaze (both partners are looking at each other) are emphasized in bold (blue and red for male and female respectively). As can be seen, during times of mutual gaze the gamma of both partners oscillates synchronously.

*Gaze*: Overall, both couples and strangers spent more time in social gaze than in no gaze during the interaction (including mutual gaze, only female looking, or only male looking); couples; M = 93%, SD = 2.08, Strangers; M = 78.66%, SD = 1.66. Yet, couples spent a greater percentage of the interaction looking at each other compared to strangers, *t* (two-tailed) = 19.86, p = 0.0001, lending support to our third hypothesis (Fig. 2).

*Affect*: Couples and strangers spent approximately half of the time expressing positive affect (including mutual positive affect, only female positive affect, or only male positive affect); M = 56.38%, SD = 3.1102, M = 55.00%, SD = 3.1240, respectively. However, in contrast to our hypothesis, no group difference was found for affect, *t* (two-tailed) = 0.71, p = 0.6089 (Fig. 2). Percentages of gaze and affect behavior in males and females in the couples and strangers groups appear in Supplementary Table 2.

#### Speech: Duration and Content

*Speech time*: Total speech time was similar in the two groups; couples: M = 73.54%, SD = 0.2406, strangers: M = 73.422%, SD = 0.2953. This suggests that the neural synchrony found in couples did not result from more talking in this group.

*Conversation content:* No difference between groups was found in the content of the conversation (see below Method). Among couples, 11 couples engaged in *practical* discussion that consisted of practical plans and considerations (45.8%), conversation in 8 couples was of the *emotional* type and involved the expression of feelings and desires (33.3%), and 5 couples engaged in a *reminiscent* conversation that focused around sharing past memories (20.8%). Among strangers, 12 pairs engaged in a *practical* conversation (48%), 8 pairs in an *emotional* conversation (32%), and 5 pairs in a *reminiscent* conversation (20%). No differences in the content of conversation was found among groups (x^2^ = 0.011, p = 0.97), suggesting that the difference in neural synchrony between couples and strangers was not driven by differences in the general conversation content.

### Connecting Behavioral and Neural Synchrony

To address the integration of behavioral and neural synchrony, we focused only on the frequency band that showed significant correlation between the brain activations of the two partners - gamma frequency localized to temporal-parietal regions. Our goal was to assess the relationship between key nonverbal social cues (gaze and affect) and neural synchrony. Using fine grained coding we analyzed the participants’ behavior-based neural synchrony, computed as the frequency-specific (gamma) power averaged across the temporal/parietal electrodes sites during episodes of gaze and positive affect as compared to episodes of no gaze and neutral affect. As dyads differed in the frequencies of various behaviors (e.g. some expressed more positive affect than others), we excluded from the groups’ Spearman correlation analysis dyads that did not meet the threshold of 6 seconds per behavior (partners that did not look at each other for at least 6 seconds throughout the entire interaction) in order to provide a sound representation of the dyadic style (see EEG preprocessing and Supplementary Table 1 for details on the number of dyads for each behavioral analysis). Figure 2 (lower panel) provides an example of one dyad’s continuous gamma power and highlights in boldface episodes of mutual gaze.

Before testing the relationship between gaze and affect and gamma synchrony, we performed an ANOVA with group (couples, strangers) and condition (gaze, affect, none) as the between-subject factors. Main effects were found for group, F (1,148) = 12.64, p = 0.00, and condition, F (2,148) = 7.48, p = 0.02, pointing to the effect of these key nonverbal behavior on the degree of gamma synchrony. Figure 2 presents mean gamma synchrony during episodes of gaze versus no gaze and during episodes of positive versus neutral affect in the couples and strangers groups.

Next, the correlation scores for gaze and affect were averaged into a single score for each group (couples, strangers), and neural synchrony during episodes of gaze and positive affect vs. episodes of no gaze and neural affect were compared for each group separately. As predicted, significant difference between moments of gaze versus no-gaze in gamma synchrony was found for couples; t (two tailed) = 2.03, p = 0.05, (couples gaze; M = 0.2156, SD = 0.0374; couples no gaze; M = 0.0480, SD = 0.0693), suggesting that gamma synchrony was higher during moments of social gaze and lending support to our fourth hypothesis. Brain-to-brain gamma synchrony during episodes of positive affect compared to neutral affect was marginally higher in the couples group, t (one tailed) = 1.57, p = 0.065 (couples positive affect; M = 0.1565, SD = 0.051; couples neutral affect; M = 0.084, SD = 0.048). This marginal effect should be treated with caution and tested in future studies. In the strangers group no significant difference was found in gamma neural synchrony between moments of gaze versus no gaze or positive affect versus no affect (Fig. 2C,D) (strangers gaze M = 0.0368, SD = 0.0447; strangers no gaze M = 0.0879, SD = 0.0712, strangers positive affect M = −0.0307, SD = 0.055; strangers neural affect M = 0.0337, SD = 0.0425). Comparison between strangers and couples revealed significantly higher gamma correlation in couples during episodes of gaze; t (two tailed) = 3.07, p = 0.0036, and positive affect, t (two tailed) = 2.53, p = 0.015 (Fig. 2E,F).

### Strangers; Social involvement, social behavior, and brain-to-brain neural synchrony

Given that neural synchrony was anchored in episodes of gaze and positive affect only among couples, we explored factors that may be associated with neural synchrony between unfamiliar male and female adults.

**Figure 3:**
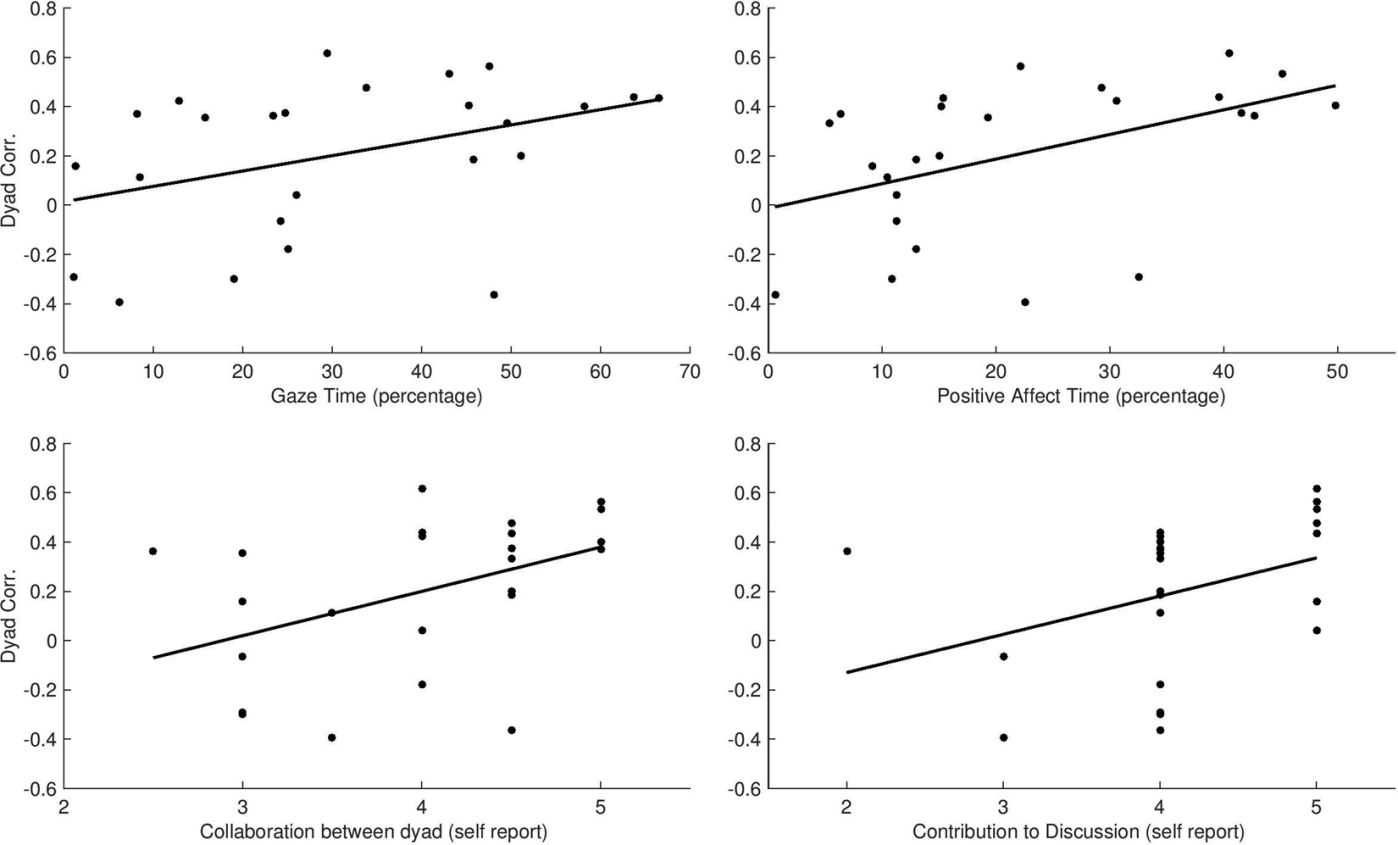
Behavioral and Neural Correlational analysis in Strangers. (**A**) Correlational analysis of gamma synchrony and length of gaze duration. Scatter plot between the strangers’ inter-brain gamma power correlation and duration of social gaze (n = 25 dyads). (**B**) Scatter plots between the strangers’ inter-brain gamma power correlation and duration of positive affect (n = 25 dyads). (**C**) Scatter plots between the strangers’ inter-brain gamma power correlation and self-reported post interaction collaboration scores (n = 24 dyads). Note, this correlation did not survive FDR correction.

Following the interaction, participants answered four questions (see Methods) that assessed their subjective experience of their own involvement in the conversation in terms of collaboration, pleasantness, and contribution to the interaction. Among strangers, but not among couples, gamma synchrony correlated with the averaged dyadic score on collaboration; R = 0.5140, p = 0.01; however, this effect did not survive FDR correction and should be treated with caution and guide future research (Fig. 3C). Females’ self-perceived contribution to the discussion correlated with the dyad’s gamma synchrony; R = 0.5313, p = 0.009 (Fig. 3D). Supplementary Table 3 presents correlations for the four questions for males and females in the couples and strangers groups.

While the strangers’ averaged gamma synchrony did not show significant differences between moments of gaze versus no gaze or positive affect versus no affect, we found correlation between the amount of time strangers spent in social gaze and their gamma synchrony, R = 0.431, p = 0.0323, and similar correlation emerged between the amount of time strangers spent in positive affect and gamma synchrony, R = 0.566, p = 0.0031 (Fig. 3A,B, respectively). These correlations did not reach significance in the couples group.

## Discussion

Social neuroscientists have advocated the need to formulate a two-person perspective on brain functioning that accommodates the inherently social nature of the human brain and its dynamic tuning to the brain of others^6^; yet the mechanisms supporting brain-to-brain coordination are not fully clear. This is the first study to utilize hyper-scanning EEG integrated with micro-level analysis of core social behavior to explore neural synchrony during naturalistic interactions. We found that during natural social moments the brain rhythms of two interacting adults showed a temporally-coupled pattern. Such neural synchrony was higher in romantic couples compared to strangers, indicating that human attachments may play a role in brain coordination and the degree of social connectedness among partners impacts brain coordination. We further found that brain-to-brain synchrony localized to temporal-parietal regions and expressed in the fast-paced gamma rhythm. Brain-to-brain gamma coupling was anchored in nonverbal social behavior; it was higher during moments of social gaze and marginally higher when individuals expressed positive affect. Gaze and affect mark the first nonverbal social behaviors mastered by infants and are used by parents to form the first human social dialogue. We focused on gaze and affect in light of the proposed continuity between the parent-child and romantic attachments and in light of models indicating that experiences within the parent-infant bond prepare the brain for social connectedness^3^. Finally, neural synchrony was independent of speech duration and general conversation content, suggesting that brain coordination may be supported by the non-verbal rather than verbal aspects of social interactions. Overall, our findings suggest that brain coupling may be anchored in the first nonverbal signals that humans learn to use, raise the possibility that brain-to-brain synchrony localizes to temporal-parietal regions, and highlight the role of attachment and social connectedness in the coordination of two brains.

Brain coupling was localized to the temporal-parietal area, covering a subset of brain regions previously implicated in embodied simulation and mentalizing functions, including the TPJ, pSTS, and IP. These partially overlapping structures are involved in social-interactive processes, such as social gaze^39,41^, social comprehension^42,43^, and self-other differentiation^41^, and studies of brain-to-brain synchrony using a variety of methods, including hyperscanning EEG, dual fNIRS, or dual fMRI, have all found neural coordination in these areas. Our findings are consistent with these studies and add the novel angle that two-brain coordination in these areas expresses specifically via gamma rhythms. Gamma real-time neural coupling was observed only in long-term couples, not in strangers, indicating that affiliative bonds may propagate neural synchrony and consistent with prior studies which showed that various indices of social connectedness, such as collaboration or predictability, augment neural synchrony^29,31^. Attachment relationships provide the most salient context for human social connectedness and include multiple relational aspects that enhance such connectedness. For instance, couples’ interactions may reverberate previous experiences^44^, be more predictable^45^, and involve interpersonal familiarity^46^, all of which have been shown to induce a greater sense of connectedness and, as seen here, lead to higher neural synchronization.

Our findings suggest that social gaze plays a key role in neural synchrony. Couples looked at each more during the interaction and among couples neural synchrony was anchored online in moments of social gaze. It is thus possible that episodes of social gaze provide a framework for the emergence of neural synchrony. Recently, Hirsch and colleagues^47^ measured brain-to-brain synchrony using fNIRS signals acquired during eye-to-eye contact between partners (similar to our paradigm) compared to a condition when both partners look at a picture of a face. Results showed greater neural synchrony in the eye-to-eye condition compared to the joint attention to a picture face in temporal and parietal areas, consistent with the current findings. Importantly, while both social gaze and positive affect characterize the first social dialogue between human parents and infants, only social gaze is universally observed while the display of positive affect is culture specific^48^. Thus, social gaze may be a species-typical early social signal that carries a profound effect on the maturation of the social brain and orients it to tune with the brains of other social beings. When the ability for social gaze is impaired, for instance, among individuals with autism spectrum disorders, functioning of the social brain is similarly impaired^49^ and future research is required to examine whether ASD is accompanied by impairments in brain-to-brain coordination during natural social interactions.

Interestingly, the neuropeptide oxytocin, which is known to increase social gaze^50^ and sense of social connectedness^51^, also augments behavioral synchrony and is increased in romantic couples compared to strangers^28,52^. Oxytocin administration has been shown to enhance brain-to-brain coordination in termporo-parietal regions during social collaboration^33^. It is thus possible that one neurobiological mechanism by which long-term attachments enhance brain-to-brain synchrony is via the increased functionality of the oxytocin system sustained by romantic relationships, the greater amount of social gaze during daily interactions, and the sense of interpersonal connectedness, comprising the neuroendocrine, behavioral, and mental levels of a long-term affiliative bond. Yet, much further research is needed to test this hypothesis. The evolution of the social brain in primates, which carried important survival function by tuning the brain to the social world^4^, may have matured within attachment relationships and culminated in the human brain’s capacity to coordinate neural response with that of social partners during moments of eye-gaze coordination to increase survival, promote safety, and enhance group cohesion.

Our finding that gamma synchrony in tempro-parietal regions is anchored in episodes of social gaze and positive affect accords with research on mother-child neural synchrony. A recent MEG study that exposed mothers and their 9 years-old children to a video of their own interaction compared to unfamiliar interaction found that own interaction enhanced gamma-band activations in the pSTS of both partners and that these gamma activations in the temporal cortex were synchronized between mother and child. Similar to the current findings, neural synchrony between mother and child was found during moments of shared gaze and positive affect but not during episodes of no gaze and neutral affect^53^. It is thus possible that gamma rhythms in temporal regions chart one mechanism by which attachment partners tune their brain for dyadic coordination in order to support and enhance the affiliative bond.

Social connectedness among couples may include not only more social gaze but also elements of predictability and familiarity, which may lead to rapid encoding of motor^54^ and communicative^55^ signals. For our couples, who have been in a committed relationship for some time, such familiarity can lead to greater neural synchrony, when familiar actions^56^ and communications^57^ are incorporated as neural predictions and pave the way for greater brain coordination. Schilbach and colleagues^6^ argue that social interactions always involve historicity and social phenomena must be understood in the context of past encounters and future trajectories. As the certainties increase during social interactions, behavioral and neural coordination may increase in parallel. Thus, our findings may suggest that human bonds are based on the establishment of a mutual neural grounding that gives rise to more precise predictions and greater certainties, creating a sense of connectedness that affords the “secure base” of attachment relationships^58^. In this context, it is of interest that as men’s attachment anxiety increased, gamma synchrony decreased in parallel, suggesting that neural coupling may be associated with the degree to which men feel connected and secure about the relationship. Importantly, while we suggest that more social connectedness is reflected in higher neural synchrony, our study did not tease apart the specific components of social connectedness, such as familiarity, salience, safety, collaboration, history, or predictability and the current study is only a first step in this direction. Much further research is needed to understand which elements in human social connectedness enhance or impede the expression of two-brain coordination.

Overall, our findings demonstrate the existence of neural synchrony during natural social interactions. We found that neural synchrony among two human adults is localized to the temporo-parietal area and expresses in gamma rhythms. We further show that social behavior in nonverbal channels that mark the first human exchange –social gaze and positive affect – contribute to social synchrony, albeit the findings for positive affect were marginal and require further research. Studying the integration of behavioral and neural synchrony in other social attachments, including parents, friends, therapists, or mentors, is an important next step and understanding when and under what conditions strangers become familiar enough to express neural synchrony requires much future research. Similarly, untangling human social connectedness to its specific components and understanding how collaboration, familiarity, predictability, and safety/danger (e.g., in-group out-group encounters) may shape the degree of neural synchrony needs much further research and our findings provide but a first step. Formulating a detailed two-person neuroscience perspective, understanding how the brain is grounded in the social world, and defining how two humans can tune their brain to each other online is an exciting new area for empirical research and theory building.

Finally, several study limitations merit consideration. First, while our goal was to use hyper-scanning EEG during an ecologically-valid naturalistic interaction, such setting affords less exact localization of brain regions as compared to fMRI or MEG scanning and enables less exact modeling of the specific task parameters. Research in social neuroscience must always oscillate between ecological validity and experimental control and both types of studies are needed to fully understand the neural basis of real-life social phenomena. The mechanisms detected here in a naturalistic context may provide a first step for future research involving more controlled experiments. Second, the reduced accuracy of EEG source localization did not enable us to pinpoint the exact regions of activation and while LORETA^59,60^ is thought to provide adequate regional estimation, the exact brain regions implicated in neural synchrony should be further tested using tools with greater spatial resolution, such as fMRI. Since research on two-brain coordination using double fMRI has its own limitations (e.g., unnatural settings, poor temporal resolution), convergent findings from multiple methodologies are needed to advance our understanding on the online coordination of two brains. Inclusion of a third group to control for familiarity (male and female friends who are not romantically involved), albeit extremely difficult to recruit while keeping familiarity constant (i.e., couples and friends matched on period of familiarity), could have differentiated findings linked to familiarity from those associated with romantic love. Our findings may provide a first step in pinpointing the oscillatory band and general brain area that support neural synchrony and may stimulate experimentally-controlled studies that can further examine how brain coupling is grounded in behavioral synchrony, how affiliative bonds shape neural synchrony, how social connectedness links with brain connectedness, and how natural social moments express in the brain as a shared experience of two interacting humans.

## Materials and Methods

### Participants

One-hundred-and-four healthy young adults participated in this study. These included 52 male-female pairs in two groups; the “couples” group included cohabitating romantic partners in a long-term relationship and the “strangers” group included unfamiliar man and women. Three dyads were excluded from further analyses since one partner’s inter-electrodes correlation did not reach the threshold of R > 0.5 (see EEG preprocessing). All participants were healthy, with no prior physical or mental illness, completed at least 12 years of education, and had no current psychopathology. The final “couples” group included 24 heterosexual couples (48 participants) who were romantically involved for at least one year. Couples were together on average 2.7 ± 1.7 years (age 25 ± 4.1 years, 13.5 ± 1.9 years of education). The “strangers” group included 25 males and 25 females who did not meet prior to the experiment and the study was their first social encounter (age 24 ± 3.6 years, 13.4 ± 1.7 years of education). To ensure unfamiliarity, strangers were separated by a screen during EEG preparation so that their first encounter occurred during the experiment. Exclusion criteria included medication intake, physical or psychiatric condition, and self-reported health problems. The study was approved by the ethical committee of Bar-Ilan University and all participants signed an informed consent. All procedures were explained to the participants before the study and were performed in accordance with ethical guidelines.

### Procedure

Participants were recruited via the internet and by ads posted in a university campus and surrounding area. Prior to arrival at the lab, participants completed self-report measures related to demographic and health information (e.g., weight, height, smoking, medication, and use of conraceptives). Experiments were conducted in a laboratory during the mid-afternoon hours (4:00–7:00 PM). A 32-electrode cap was placed on each participant’s scalp and the forearm of their non-dominant hand was attached to the chair handle to restrict arm and neck movements. The first paradigm included a 3-minute rest with eyes open while the screen was still standing between the participants. Next, participants sat next to each other in a 3-feet distance between their faces while facing each other in a 45-degree angle. This position enabled partners to look at each other during the interaction and for their facial and bodily signals to be captured by a camera on an adjacent wall. Participants were asked to sit comfortably and engage in a positive interaction (“fun day” paradigm) for five minutes. The paradigm involves planning a fun day to spend together and has been previously validated at our lab^28,61^. Interactions were videotaped for later offline coding. After the interaction, participants completed four questions related to their feeling about the interaction (see below). Participants received 50 USD for participation.

### Self-reported Measures

Participants completed questionnaires using the online platform www.qualtrics.com. This included demographics questionnaires and the Revised Experiences in Close Relationships (ECR-R), a self-report measure of romantic attachment. The ECR-R assesses attachment along two orthogonal dimensions; anxiety about the relationship and avoidance of intimacy, and a high score on each reflects insecure attachment^62^.

### Dual-EEG data acquisition

Neuroelectric activity in the two participants was simultaneously and continuously recorded while they were engaged in the interaction. The system was composed of two Acticap helmets with 32 active electrodes arranged according to the international 10/20 system including one electrooculography (EOG) electrode and referenced to the common vertex (Cz), with analog 0.1–500 Hz band-pass filtering. The impedances were maintained below 10 kV. Data acquisition was performed using a 64-channels Brainamp amplifier from the Brain Products Company (Germany) to enable the computation of millisecond-range synchrony between the two EEG recordings^63^.

### Social Interaction Behavior Analysis

Coding was conducted offline by coders trained to reliability who were blind to all other information. We used a microlevel second-by-second coding scheme previously validated in our lab and consistent with prior research that showed correlations between these micro-level behaviors and brain activations^64^. Coding was conducted for the first 3 minutes of the interaction consistent with our prior research^14^.

#### Micro-coding of social synchrony

Gaze and affect, the main non-verbal channels of social communication, were coded using a set of mutually-exclusive codes consistent with our prior brain and behavioral research^20,64^. Coding for the two partners was conducted in separate passes using a computerized system (Noldus, Waggenigen, The Netherlands) while the system was set to 0.01 s accuracy. The following codes were used for each participant:

*Gaze* –

Social gaze – Looking at partner’s face

Gaze to object – Looking at an object in the environment (including, for instance, partner’s legs)

Gaze aversion – Looking away from partner’s face but not focusing on any object

Here we use the term Social Gaze to denote looking at the partner’s face and No Gaze, to denote gaze at object or gaze aversion (i.e., no social gaze).

*Affect* –

Positive - Clear expressions of high positive arousal or energy indicated by laugh, giggle, excited talk, or positive excitement

Neutral – No clear expression of any specific affect. Facial expression is pleasant/neural and arousal is low

Negative: Withdrawn – Clear expression of negative affect. Facial expression is sad or withdrawn, facial expression is flat, body position/muscle tone express disengagement.

Negative: Angry - Negative arousal is clearly indicated by angry voice, screams, scolding, scary or angry body movement or looming.

*Speech* – speech, no speech.

Inter-rater reliability was computed on 20 interactions and inter-rater reliability exceeded 90% on all codes (kappa = 0.87, range = 0.81–95).

Speech Content: The task of the conversation was to plan a fun day to spend together. Using the well-validated CIB coding scheme for adult-adult interactions^65^, we coded interaction content into three groups (a*) Practical* - Partners were dealing with the task in a rational and practical manner (e.g., “we should do x and then y”, if we do x we won’t have time for y”). (b) Emotional – Partners mainly focused on expressing emotions (e.g., “I really like to do that”, “this gets me very excited”, “this is really disappointing”). and (c) Reminiscent – partners mainly shared memories of past positive experiences, places, or activities they did and wish to do again.

Coding was conducted by two individuals who trained to use the CIB coding system and reliability on 20 interactions averaged 93% (intraclass r = 0.93).

### EEG preprocessing

Matlab (Mathworks Inc, Natick, MA), EEGLAB^66^ and Fieldtrip toolbox for MATLAB^67^, were used for all calculations. The continuous EEG data was low-pass filtered with a cutoff of 60 Hz to reduce motor artifacts, and Spatial Independent Component Analysis (ICA) was applied in order to clean eye movements and blinks. A digital notch filter was applied at 50 Hz and its harmonics to remove artifacts caused by alternating current line noise. Data from three couples were omitted from analysis due to low inter-correlation among relevant electrodes and the final sample comprised 98 individuals (couples = 24 dyads, strangers = 25 dyads).

### Extended gamma related EMG artifacts removal

A possible confounding effect for cortically induced gamma power is electromyographic (EMG) activity from scalp and neck muscles^68^. In addition to the common source of noise in EEG experiments, the free conversation setup can naturally impose ‘electrode drift’, the physical movement of the electrode relative to the brain or different levels of electrodes detachment from the head. In this study, we applied three methods to reduce EMG noise from the signal. First, we visually inspected the raw data, extracting artifact-free epochs, reducing large muscle artifacts^69^ (see EEG Frequency Analysis). Second, in each hemisphere, we used only electrodes that at least moderately correlated with each other (R > 0.5) to create a coherent and stable mutual signal, reducing large and small muscle artifacts. Averaging the electrodes and applying the threshold improve signal quality and reduce noise from non-cerebral sources. The threshold of R = 0.5 was chosen since Spearman Correlation ranks with magnitudes higher than 0.5 are considered moderate and above^70^ We set the lower threshold of gaze to 6 seconds of gaze during the entire interaction based on previous finding showing that a single gaze during typical conversation lasts on average 4 and 7 seconds for males and females respectively^71^. When the cumulative amount of gaze during an entire interaction does not reach this amount, which characterizes a single gaze, this may point to atypical interaction^72^. We coded episodes of speech versus non-speech during the interaction to control for large and small muscle artifacts.

### EEG Frequency Analysis

We conducted two separate frequency calculations; (1) continuous: for the rest and the social interaction paradigms and (2) epochs-based analyses: only for the social interaction paradigm. The continuous calculation was performed on the 3 minutes of rest and the 5 minutes of the conversation and used to estimate synchronous neuro-electrical activity between dyads. The epoch-based frequency calculation was performed on the first 3 minutes of the experiment, consistent with prior micro-analytic studies^14^, and was used to anchor the neural synchrony in episodes of social gaze and positive affect.

An additional noise removal process was conducted separately for each frequency calculation. For the epoch-based frequency calculation noisy epochs were visually inspected and manually extracted as described above and for the continuous frequency calculation EMG artifacts were identified and removed using independent component analysis (ICA) based on their characteristic topographies, time courses, and frequency distributions^73^. For both calculations and for each electrode, the absolute spectral power was grouped into frequency bands: theta (4–8 Hz), alpha (8–12 Hz), beta (12–30 Hz) and gamma (30–60 Hz), with no overlap between frequencies during the analysis. Electrodes were collapsed into four Regions of Interest (ROI): frontal (F3,F4,F7,F8), parietal (P3,P4,CP1,CP2), temporal/parietal (T7,T8,P7,P8,CP5,CP6), and occipital (O1, O2) and the spectral power was calculated for each ROI separately.

### EEG Continuous frequency calculation

Time frequency representation of the continuous EEG (over the full 3 minutes of rest and 5 minutes of the social conversation) was calculated using the Stockwell transform^74^ with a time resolution of 0.002 sec and a frequency resolution of 0.3 Hz. Two analyses were performed: **(**a) Dyadic continuous spectral EEG synchronous calculation; and (b) Behavioral and temporal/parietal gamma correlation.

- **Dyadic continuous spectral EEG Synchronous calculation**. The brain-to-brain neural synchrony was quantified using the Spearman correlation between the two partners’ spectral power and power was computed over the entire social interaction (300 Sec). The Spearman correlation was computed over time signal of the Stockwell transform frequency spectrum (for each frequency bins in the range of 4–60 HZ), averaged over each ROI electrodes (frontal, parietal, temporoparietal, occipital) in the two partners. Dyadic correlation values per bin and per ROI were averaged across groups (couples, strangers). Significance of the results was evaluated using t-test over the averaged of the correlation values grouped into the four main frequencies bands (theta, alpha, beta, and gamma). FDR correction was applied to the resulting correlation p values of all comparisons and tested at 0.05 level^75^.
- **Behavioral and temporal/parietal gamma correlation analysis**. The dyadic gamma temporal/parietal correlation value was correlated with the corresponding behavioral data, including questionnaires, gaze, and affect variables. First, we performed an ANOVA with gamma synchrony as the dependent variable and group (couples vs strangers), and condition (gaze, affect, none) as the between-subject factors. Following, t-tests explored differences between groups’ averaged values (see Results section for data on group and Fig. 2 for data on condition). FDR correction was applied to the resulting correlation p values of comparisons and tested at 0.05 level^75^.

### EEG Epoch’s frequency calculation

Data were segmented into 1000 ms. epochs. A Hamming window was used to control for artifacts resulting from data splicing. Trials containing power jumps and/or muscle artifacts were visually rejected. The frequency calculation was done on each of the remaining trials, down-sampled to 5HZ for each trial separately.

### Dyadic Segmented Behavioral and EEG Synchronous calculation

The behavioral and brain synchronization between partners were evaluated in terms of the correlation of EEG oscillatory amplitudes in specific frequency bands and times of relevant behavior, respectively. Specifically, behavioral measures of specific gaze and affect patterns were defined and Spearman correlation was computed between each dyad’s temporoparietal gamma power during these time segments. Next, t-tests were applied to assess the significance of the differences between the groups’ gamma correlation scores during times of gaze and positive affect.

### Rest Analysis

To compare the dyadic temporal-parietal gamma synchrony during the social interaction and the rest experiment, the same steps of dyadic continuous calculation were repeated over the rest signal of the participants limited to the temporoparietal ROI and the gamma frequency. Next, t-tests were applied to assess the differences between gamma correlation scores during rest and during social interaction in each group.

### Post-Interaction Questions

Following the interaction male and female participants answered the following questions on a five-point Likert scale from 1 (low) to 5 (high)

1. What was the level of collaboration between you and your partner during the interaction?
2. How pleasant was the interaction between you and your partner during the interaction?
3. How much was your partner’s contribution central to the interaction?
4. How much was your contribution central to the interaction?

## Electronic supplementary material

Supplementary information
