# Supplementary material for: Brain-to-Brain Synchrony during Naturalistic Social Interactions
Source: Sci Rep. 2017 Dec 6;7:17060. doi: 10.1038/s41598-017-17339-5 (PMC5719019; doi:10.1038/s41598-017-17339-5)
Supplement: Supplementary file 1 — Supplementary information [file 41598_2017_17339_MOESM1_ESM.pdf]

Brain-to-Brain Synchrony during Naturalistic Social Interactions

Sivan Kinreich[1]

Amir Djalovski[2]

Lior Kraus[1]

Yoram Louzoun[1,3]

Ruth Feldman[4,5]

[1]Gonda Brain Sciences Center, Bar-Ilan University

[2]Department of Psychology, Bar-Ilan University

[3]Department of Mathematics, Bar-Ilan University

[4]Baruch Ivcher School of Psychology, Interdisciplinary Center, Herzlia

[5]Yale University Child Study Center

For Correspondence

Ruth Feldman, PhD

Simms-Mann Professor of Developmental Social Neuroscience

Baruch Ivcher School of Psychology,

Interdisciplinary Center (IDC) Herzliya,

P.O. Box 167, Herzliya 46150, Israel, Phone: 972-544566353

email: feldman.ruth@gmail.com

Running Title; Brain-to-Brain Synchrony

Supplementary Table 1: Summary of trials and electrodes included in the analysis (%)

	Strangers %Mean (SD)	Couples %Mean (SD)	*t*-test
# Trials	70.12 (15.48)	73.67 (13.2)	p< 0.34
# Electrodes	73.14 (5.02)	71.92 (4.66)	*p*<0.792

Supplementary Table 2. Percentages of Dyadic behaviors in Males and Females Couples and Strangers

Groups	Behavior	Both<br>% Mean (SE)<br>[N]	Female<br>% Mean (SE)<br>[N]	Male<br>%Mean (SE)<br>[N]	Neutral<br>% Mean (SE)<br>[N]
Couples	Positive Affect	26.73 (3.97)<br>[21]	18.5 (2.93)<br>[21]	11.12 (2.42)<br>[21]	41.42 (8.76)<br>[19]
	Gaze	52.81 (2.91)<br>[24]	20.59 (1.71)<br>[24]	20.07 (1.61)<br>[24]	6.51 (2.96)<br>[6]
Strangers	Positive Affect	23.79 (3.26)<br>[23]	16.04 (4.11)<br>[23]	15.16 (1.98)<br>[23]	42.68 (5.92)<br>[20]
	Gaze	28.65 (1.59)<br>[25]	23.08 (1.6)<br>[25]	26.93(1.79)<br>[25]	21.33 (6.31)<br>[11]

Supplementary Table 3: Correlations between Gamma Synchrony and the Dyads' Subjective Experience

Subjective Assessment	Men stranger	Women stranger	Men couple	Women couple
1.Collaboration between the dyad	R=0.43<br>P= 0.03 *	R=0.47<br>P= 0.01 *	R=0.24<br>P= 0.26	R=-0.04<br>P= 0.83
2. How pleasant was the interaction	R=0.36<br>P= 0.07	R=0.07<br>P=0.74	R=0.43<br>P= 0.04 *	R=0.17<br>P= 0.41
3. Contribution of the partner to the interaction	R=0.30<br>P= 0.15	R=0.15<br>P= 0.46	R=0.58<br>P= 0.00 **	R=-0.12<br>P= 0.57
4. Contribution of yourself to the interaction	R=0.28<br>P= 0.17	R=0.53<br>P= 0.00 **	R=0.14<br>P= 0.51	R=0.16<br>P= 0.46

R – Spearman correlation

* p<0.05

** p<0.01

Power Calculations

FDR correction was applied to the resulting correlation p values of (n= 8; groups and brain lobes * frequencies bins) comparisons and tested at 0.05 level [75], and Cohen's effect size [76] was calculated over all the correlation results. The cortical sources of the couples EEG rhythms within a given frequency range[39] were estimated by low-resolution brain electromagnetic tomography (LORETA)[77–79],
